# Psychological Features Associated With Awake Bruxism in Painful TMD: The Role of Anxiety

**DOI:** 10.1111/joor.70158

**Published:** 2026-01-28

**Authors:** Dyanne Medina Flores, Samilla Pontes Braga, Giancarlo De La Torre Canales, Juliana Stuginski‐Barbosa, Paulo César Rodrigues Conti

**Affiliations:** ^1^ Department of Prosthodontics, Bauru School of Dentistry University of São Paulo São Paulo Brazil; ^2^ Division of Oral Rehabilitation, Department of Dental Medicine Karolinska Institutet Huddinge Sweden; ^3^ Egas Moniz Center for Interdisciplinary Research (CiiEM), Egas Moniz School of Health & Science Almada Portugal; ^4^ Bauru Orofacial Pain Group Bauru SP Brazil

**Keywords:** anxiety, awake bruxism, depression, pain catastrophizing, perceived stress, temporomandibular disorder

## Abstract

**Background:**

Awake bruxism (AB) is closely linked to psychological factors and commonly co‐occurs with painful temporomandibular disorders (TMD).

**Objective:**

To evaluate the role of anxiety comparing the frequency of AB behaviours, as well as levels of perceived stress, pain catastrophizing and depressive symptoms among patients with painful TMD, categorised by clinical anxiety levels.

**Methods:**

A total of 72 patients diagnosed with painful TMD were enrolled and classified into two groups based on *T*‐scores derived from the Generalised Anxiety Disorder 7‐item scale [GAD‐7]: elevated symptoms of anxiety group (*T* ≥ 61): 30 and normative anxiety (*T* ≤ 60): 42. *T*‐score calculations were based on a previously studied pain‐free control group. AB behaviours were recorded through Ecological Momentary Assessment (EMA), and participants completed the Perceived Stress Scale [PSS], the Pain Catastrophizing Scale [PCS] and the Patient Health Questionnaire‐9 [PHQ‐9], alongside lifestyle assessment. Between‐group comparisons and correlation analyses were conducted to evaluate associations between anxiety and clinical, behavioural and psychosocial outcomes.

**Results:**

Patients with elevated symptoms of anxiety exhibited higher total AB frequency (89%; 72.12%; *p* < 0.002), particularly increased tooth clenching (26.90%; 13.48%; *p* < 0.016). They also reported significantly higher clinical pain intensity (*p* < 0.005), as well as elevated perceived stress (*p* < 0.001), higher pain catastrophizing (*p* < 0.032), especially helplessness (*p* < 0.050) and rumination (*p* < 0.47) and more severe depressive symptoms (*p* < 0.002). No significant differences were seen between groups in physical activity, social engagement, alcohol drinking or smoking.

**Conclusion:**

These findings underscore that TMD patients with elevated clinical symptoms of anxiety exhibit significantly higher frequencies of AB, specifically tooth clenching, psychological distress and pain intensity.

## Graphical abstract

Objective: Evaluate the role of anxiety by comparing the frequency of AB behaviours, as well as levels of perceived stress, pain catastrophizing and depressive symptoms among patients with painful TMD, categorised by clinical anxiety levels. Methods and Study Protocol: TMD anxiety subgroups (*N* = 72): Clinical elevated anxiety symptoms (*T* ≥ 61) *n* = 30. Clinical normative anxiety (*T* ≤ 60) *n* = 42. Validated questionnaires for signs and symptoms of anxiety [GAD7], stress [PSS], depression [PHQ‐9] and pain catastrophizing [PCS] were collected. Participants underwent AB frequency assessment via EMA using a smartphone that sent alerts at random intervals every day for 1 week. They reported in real time which of the AB behaviours best represented their current condition: relaxed jaw muscles, tooth contact, tooth clenching, tooth grinding or jaw bracing. Statistical Analysis: Descriptive statistics of behaviours, including quantitative measures and categorical variables, were performed. Mann–Whitney was employed for group comparisons. Data from the control group reporting over 85% relaxed jaw muscles were used to transform the total score of GAD7 results into *T*‐scores. Transformed into *z* score = (value for a single patient—mean of controls/SD of controls), then the formula *T* score = 10(*z* score) + 50 was applied. Subsequently, TMD patients were divided into patients with clinically elevated anxiety (*T*‐score ≥ 60) and those with normative anxiety symptoms (*T*‐score ≤ 60). Key Findings: Increased Total AB 89.08% in the clinically elevated anxiety group, particularly teeth clenching 26.9%. Clinical pain intensity (CPI) increased in the clinically elevated anxiety group versus the normative group. Elevated perceived stress (*p* < 0.001), higher pain catastrophizing (*p* < 0.032), especially helplessness (*p* < 0.050) and rumination (*p* < 0.47) and more severe depressive symptoms (*p* < 0.002). Conclusions and Clinical Relevance. Anxiety plays a key role in the expression of AB behaviours and the psychosocial profile of patients with painful MD. Notably, tooth clenching emerged as the characteristic behaviour in painful TMD patients with elevated anxiety symptoms. Therefore, elevated anxiety symptoms should be considered a clinical target in the management of AB behaviours in TMD patients.

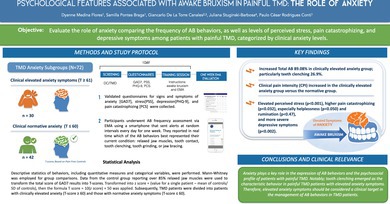

## 1 Introduction

An anxiety state is characterised by subjective feelings of tension, apprehension, nervousness and worry, in preparation for future danger and cautious or avoidant behaviours, along with activation (arousal) and discharge of the autonomic nervous system [1]. Anxiety states can be distinguished from other unpleasant emotions such as anger, sorrow or grief, by their unique combination of experiential, physiological and behavioural manifestations [2]. The role of emotional factors, as anxiety, has been highlighted in the literature as contributing to the onset and persistence of bruxism and temporomandibular disorders (TMD) [3, 4, 5, 6].

TMDs encompass a heterogeneous group of musculoskeletal conditions affecting the masticatory muscles, the temporomandibular joints (TMJ) and related structures, representing one of the most prevalent causes of non‐dental orofacial pain in adults [7]. Chronic painful TMD has been consistently linked to a spectrum of biopsychosocial factors, among which psychological constructs such as anxiety play a pivotal role in pain perception and maintenance [3]. Anxiety has been identified as a significant risk factor for painful TMD, with higher trait anxiety doubling the odds of diagnosis and predicting greater pain severity and muscle fatigue [8, 9, 10]. The OPPERA study revealed that chronic TMD patients exhibit greater psychological distress, including higher anxiety, depression, catastrophizing, stress and poor sleep, compared to healthy controls [11]. Moreover, these factors have been consistently associated with increased pain sensitivity, poorer clinical outcomes, and the persistence of behaviours like AB in orofacial pain populations [12, 13, 14, 15].

AB is defined as ‘masticatory muscle activity during wakefulness characterized by repetitive or sustained tooth contact and/or by bracing or thrusting of the mandible’ [16, 17]. This behaviour has been associated with muscular and articular overload, with the potential to exacerbate myofascial pain and TMJ arthralgia [18]. Consequently, the interplay between emotional states and AB may establish a vicious cycle of increased muscle tension and pain, whereby anxiety and stress act as triggers for bruxism behaviours, which in turn could amplify pain perception [19].

Although previous studies have linked psychological distress to painful TMD, few have examined how these emotional variables are related to real‐time AB behaviours in painful TMD patients. Most of the available research relies on retrospective AB self‐report measures, which limits ecological validity, whereas Ecological Momentary Assessment (EMA) offers a more reliable and valid method by capturing real‐time AB behaviours in naturalistic settings [20, 21]. Particularly, it is crucial to assess the role of anxiety in this scenario by characterising the profiles of patients according to anxiety levels, given its potential role as a central modulator of pain and bruxism [5, 22]. Therefore, this study combines the EMA assessment of AB behaviours [21], with standardised self‐report questionnaires for psychological variables. Identifying these relationships between the mentioned variables may help to clarify the mechanisms linking affective dysregulation to parafunctional activity and inform multidisciplinary strategies aiming to improve clinical outcomes in individuals with painful TMD.

Thus, this study aimed to examine the role of anxiety in real‐time reports of AB behaviours, by comparing the frequency of AB behaviours as well as perceived stress, pain catastrophizing and depressive symptoms in painful TMD individuals categorised by clinical anxiety level.

## 2 Material and Methods

The study was approved by the local ethics committee for research involving human beings of the Bauru School of Dentistry, University of São Paulo, Brazil (Certificate of presentation for ethical consideration #99729118.6.0000.5417). A signed informed consent in accordance with the Helsinki guidelines was obtained from all participants. This observational cross‐sectional study was conducted by following the recommendations of the strengthening the reporting of observational studies in epidemiology (STROBE) guidelines [23].

### 2.1 Study Sample

The sample size consisted of 122 healthy participants as a control group (81 females, 48 males; mean age: 27.7 ± 3.16 years) and 80 participants with painful TMD (52 females, 28 males; mean age: 28.60 ± 7.2 years) between 20 and 40 years old. TMD participants were divided into two groups: clinical *elevated symptoms of anxiety group* (*n* = 38) and a clinical normative anxiety group TMD (*n* = 42). Group allocation was based on GAD‐7 scores as detailed in the Statistical Analyses section. The control cohort of healthy individuals was previously recruited and characterised by the same research group and was used exclusively as a normative reference to generate standardised *T*‐scores for the GAD‐7. I Sample size calculation was performed with a software programme (G*Power 3.1.9.2; Heinrich‐Heine‐ Universität Düsseldorf). The following parameters were considered: test power of 0.8, 0.05 significance level and an effect size of 0.4.

This study was conducted in the Orofacial Pain Clinic Center at the Bauru School of Dentistry, University of São Paulo, between May 2023 and December 2024. The Brazilian Portuguese version of the Diagnostic Criteria for Temporomandibular Disorders (DC/TMD) Axis I was used for the clinical examination and diagnosis of the patients [24]. All evaluations were performed by an experienced and calibrated investigator (D.M.F). The inclusion criteria were male and female participants diagnosed with painful TMD at the established age range. Exclusion criteria included facial skin lesions, history of orofacial trauma affecting somatosensory function, acute or chronic pain conditions (e.g., migraine, fibromyalgia), systemic diseases (such as metabolic or cardiovascular disorders), neurological disturbances, current orthodontic treatment, and, for women, being outside the early follicular phase of the menstrual cycle (phase selected to minimise the potential hormonal influence on pain sensitivity and psychological outcomes).

### 2.2 Ecological Momentary Assessment

The protocol to assess AB through the EMA technique from a previous study by the same research group was applied in this study [25]. Participants received 10 alerts via WhatsApp at randomised times over five weekdays and one weekend day, prompting them to respond in real time to the question: ‘Which of the options best describes your tooth contact at this moment?’ Response options included relaxed jaw muscles, jaw bracing, tooth contact, clenching and grinding. Activities involving eating or talking were excluded. During a prior training session, participants were instructed on how to identify AB behaviours and respond to alerts, which expired after 5–15 min. Alerts were sent between 8 a.m.–12 p.m. and 2 p.m.–8 p.m., avoiding mealtimes. Data with ≥ 60% response compliance were included [26]. The frequency of AB behaviours was calculated as the percentage of affirmative responses over total valid alerts, and all AB‐related behaviours were described as total AB.

### 2.3 Clinical Variables

The following clinical parameters were collected:

- *Sex*: Categorised as male or female
- *Age*: Recorded in years at the time of assessment
- *Characteristic pain intensity (CPI)* was measured by a numerical rating scale embedded in the *Graded Chronic Pain Scale (GCPS)*, as outlined in Axis II of the DC/TMD. Participants responded to three items evaluating facial pain on a 0–10 scale: current pain intensity, the worst facial pain experienced in the past 6 months, and the average facial pain during that period. On each scale, 0 represents ‘no pain’ and 10 denotes ‘the worst pain imaginable’. Respondents indicated the number that best represents their experience. The mean of the three scores was calculated and multiplied by 10, yielding a standardised score ranging from 0 to 100 to determine overall pain intensity for subsequent analysis [27].

### 2.4 Psychosocial Variables

#### 2.4.1 Generalised Anxiety Disorder 7‐Item Scale (GAD7)

All patients completed the Generalised Anxiety Disorder 7‐item scale (GAD‐7). The GAD‐7 is a unidimensional, self‐administered questionnaire designed to screen for and quantify the severity of generalised anxiety symptoms over the preceding 2 weeks. Each of its seven items matches the fourth edition of the Diagnostic and Statistical Manual of Mental Disorders (DSM‐IV‐TR) diagnostic criteria. It assesses a distinct facet of anxiety (e.g., feeling nervous, inability to control worrying, restlessness, irritability, feeling afraid) on a four‐point Likert scale ranging from ‘not at all’ (0) to ‘nearly every day’ (3). Total scores, obtained by summing individual item responses (0–21), stratify anxiety severity into none/normal (0–4), mild (5–9), moderate (10–14) and severe (15–21) categories, with a threshold score of ≥ 8 indicating clinically significant anxiety symptoms [28].

#### 2.4.2 Perceived Stress Scale (PSS)

Perceived stress levels were measured using the *Perceived Stress Scale (PSS‐10)*. This validated self‐administered tool assesses the occurrence of stressful situations and gauges the individual's perceived ability to manage and cope with them. The scale consists of 10 items: six positively stated and four negatively stated, rated on a 5‐point Likert scale ranging from ‘never’ (0) to ‘very often’ (4). This study employed the Portuguese version of the PSS‐10, adapted for cultural and linguistic relevance [29, 30].

#### 2.4.3 Patient Health Questionnaire—9 (PHQ9)

The PHQ‐9 is a nine‐item depression questionnaire drawn from the full Patient Health Questionnaire version. It assesses the frequency, over the preceding 2 weeks, of core depressive symptoms as: depressed mood; anhedonia (diminished interest or pleasure); sleep disturbances; fatigue or low energy; appetite or weight changes; feelings of guilt or worthlessness; concentration difficulties; psychomotor slowing or agitation; and suicidal ideation. Each item is rated on a 4‐point Likert scale—0 (‘not at all’), 1 (‘several days’), 2 (‘more than half the days’) and 3 (‘nearly every day’)—yielding a total score from 0 to 27. A tenth question evaluates the degree to which these symptoms interfere with daily functioning (e.g., work, school and social activities) [31].

#### 2.4.4 Pain Catastrophizing Scale (PCS)

Psychological traits related to pain catastrophizing were assessed using the Pain *Catastrophizing Scale (PCS)*. The PCS consists of 13 items rated on a 5‐point Likert scale, designed to capture the frequency of catastrophic thoughts and emotional responses associated with pain. The total score, ranging from 0 to 52, reflects the degree of catastrophizing and includes three subscales: rumination, magnification and helplessness. The scale is organised into three distinct subdomains: hopelessness (items 8–11), magnification (items 6, 7 and 13) and rumination (items 1–5 and 12); and has demonstrated strong psychometric performance, with a robust comparative fit index of 0.98 and excellent internal consistency (Cronbach's *α* = 0.95). Total PCS score and subscale scores were used for statistical analysis [32, 33].

### 2.5 Life‐Style Variables

- *Physical activity*: Assessed via a single item classifying exercise frequency as ‘none or minimal’, ‘moderate’, or ‘high’. Moderate or high activity levels were considered physically active; those indicating minimal or no exercise were considered inactive.
- *Social activity*: Measured with a single question regarding engagement in social activity, also rated as ‘none or minimal’, ‘moderate’ or ‘high’. Moderate or extensive participation in recreational, family, or hobby activities was classified as socially active; those reporting little to no engagement were classified as socially inactive.
- *Drinking coffee*: Patients were asked using a single question about their coffee consumption frequency (no drinking, < 1–3 times/week, 1–2 times/day, > 6 times/day). Patients who reported more than < 1–3 times/week were considered positive for drinking coffee. They were considered negative if they reported no consumption.
- *Alcohol consumption*: Patients were asked using a single question about alcohol consumption frequency (no drinking, rare occasions, 1–2 weeks, > 3 weeks). Patients endorsing 1–2 weeks and > 3 weeks were considered positive for alcohol consumption. They were considered negative if they reported no drinking or rare occasions of consumption.
- *Smoking*: Assessed with a single question regarding the smoking frequency, also rated as ‘none or minimal’, ‘moderate’, or ‘high’. Moderate or high consumption of cigarettes was classified as a positive smoker status, and none or rare consumption was classified as a negative smoker status.

### 2.6 Data Analysis

Analyses were performed with a statistical software programme (IBM SPSS Statistics for MacOS, v29.0; IBM Corp). Descriptive statistics were calculated for all variables. Quantitative variables include AB behaviours (relaxed jaw muscles, jaw bracing, tooth contact, tooth clenching, tooth grinding), as well as age, pain intensity and scores from the PHQ‐9, PSS and PCS. Categorial outcomes are described in percentages (sex, TMD diagnoses, and physical and social activity). The Kolmogorov–Smirnov test assessed data for normality, and a log10 transformation was performed when the test results were significant, with an alpha level of 5% (*p* < 0.050).

Data from the control group reporting over 85% relaxed jaw muscles were used to transform the total score of GAD‐7 results into *T*‐scores based on reference data [25]. This analysis allowed us to contextualise anxiety scores within the specific variability of our population, enhancing sensitivity and interpretability compared to standard GAD‐7 cutoffs categories. Initially, patient data were transformed into *Z*‐scores with the following formula: *Z*‐score = (value for a single patient—mean of controls/SD of controls) [34]. As the *T*‐score is also a standardised score, the *Z*‐score can be transformed into a *T*‐score by the following formula: *T*‐score = 10(*z‐*score) + 50, where a *T*‐score of 50 corresponds to the control mean and each 10‐point increment represents one standard deviation. Accordingly, a *T*‐score of 50 indicates an individual value equal to the group mean of healthy, and *T*‐scores between 40 and 60 (±1 SD) define the normative range, while values above 60 indicate clinically elevated scores relative to controls. Applying these thresholds to the GAD‐7 anxiety data, we partitioned the TMD cohort into ‘normative anxiety’ (*T* ≤ 60) and ‘clinically elevated symptoms of anxiety group’ (*T* ≥ 61) groups, thereby establishing a robust framework for subsequent analyses of anxiety in relation to the other variables.

Additionally, test comparisons of the partitioned TMD groups were performed with a *t*‐test and a Mann–Whitney test. Categorical variables were explored in a chi‐square test to evaluate the association with the clinically elevated symptoms of anxiety group. Finally, a Spearman correlation for multiple comparisons was used to analyse the relationship between the principal variables and AB behaviours in both groups. A (*p* < 0.05) was considered for all the statistical analyses.

## 3 Results

### 3.1 Demographic and Clinical Variables

A total of 72 TMD patients were included in the final sample (48 women and 24 men with a mean age: 28.85 ± 7.3 years), with 30 individuals included in the clinically elevated symptoms of anxiety group (*T*‐score ≥ 61) and 42 in the normative anxiety group (*T*‐score ≤ 60). No significant difference in age was observed between groups (*p* = 0.506). However, the elevated anxiety group had a significantly higher proportion of females (83.3%) compared to the normative anxiety group (54.8%), *p* = 0.011; an odds ratio of 0.242 (95% CI: 0.077–0.75) indicates that the odds of being male were significantly lower in the elevated anxiety group, confirming that females were more likely to exhibit clinically significant anxiety symptoms. CPI was significantly higher in the clinically *elevated symptoms of anxiety group* than in the normative anxiety group (58.30 vs. 48.30; *p* = 0.005), indicating greater clinical pain severity in more anxious individuals (Table 1).

**TABLE 1:** Comparison of AB behaviours, psychological and lifestyle variables between GAD7 *T* score groups.

		TMDs with elevated anxiety (*n* = 30)	TMDs with normative anxiety (*n* = 42)	*p*
Age		26.30 (10.0)	27.54 (1.0)	0.506
Sex	Female	25 (83.3%)	23 (54.8%)	0.011^b^
	Male	5 (16.7%)	19 (45.2%)	
CPI		58.30 (1.92)	48.30 (2.08)	0.005^a^
Relaxed jaw muscles (%)		10.92 (18.0)	27.88 (25.2)	0.002^a^
Jaw Bracing (%)		21.97 (24.72)	24.78 (26.9)	0.868
Teeth Contact (%)		28.83 (28.5)	21.94 (24.5)	0.441
Teeth Clenching (%)		26.90 (20.59)	13.48 (26.49)	0.016^a^
Teeth Grinding (%)		0.0 (2.82)	0.0 (0.0)	0.147
Total AB frequency (%)		89.08 (18.0)	72.12 (25.19)	0.002^a^
PSS		32.50 (13.7)	10.00 (9.55)	< 0.001^a^
PHQ9		11.0 (9.0)	6.0 (5.75)	0.002^a^
PCS helpleness		7.0 (7.0)	5.0 (6.0)	0.050^a^
PCS magnification		4.0 (2.75)	3.0 (3.75)	0.134
PCS rumination		7.0 (8.8)	4.50 (5.75)	0.047^a^
PCS total		20.0 (13.5)	13.0 (12.75)	0.032^a^
Physical activity	Present	19 (63.3%)	28 (66.7%)	0.770
	Absent	11 (36.7%)	14 (33.3%)	
Social activity	Present	22 (73.3%)	33 (78.6%)	0.606
	Absent	8 (26.7%)	9 (21.4%)	
Coffee	Present	20 (66.7%)	25 (59.5%)	0.537
	Absent	10 (33.3%)	17 (40.5%)	
Alcohol	Present	8 (26.7%)	15 (35.7%)	0.417
	Absent	22 (73.3%)	27 (64.3%)	
Smoking	Present	0 (0.0%)	2 (4.8%)	0.225
	Absent	30 (100%)	40 (95.2%)	

*Note:* Frequency distribution, median and (IQR, interquartile range) of the variables study.

^a^ Mann–Whitney *U* test: significant differences (*p* < 0.05) between groups.

^b^ Chi quadrado test significant differences (*p* < 0.05) between groups.

### 3.2 Awake Bruxism Behaviours

Regarding AB patterns, the elevated anxiety group showed a significantly lower frequency of relaxed jaw muscle reports (10.92% [IQR: 18]) compared to the normative anxiety group (27.88% [IQR: 25.2]) (*p* = 0.002). Conversely, the total AB frequency, including all behaviours, was significantly higher in the elevated anxiety group (89.08% [IQR: 18]) than in the normative anxiety group (72.12% [IQR: 25.2]) (*p* = 0.002). Among specific AB behaviours, only teeth clenching differed significantly between groups, being more frequent in the clinically elevated anxiety group (26.9% [IQR: 20.6] vs. 13.48% [IQR: 26.49], *p* = 0.016). No significant differences were observed in jaw bracing (*p* = 0.868), teeth contact (*p* = 0.441), or teeth grinding (*p* = 0.147).

### 3.3 Psychosocial Profiles

Consistent with group categorisation, patients with clinically elevated symptoms of anxiety exhibited significantly higher scores in perceived stress (32.50 [IQR: 13.7]; 10.00 [IQR: 9.55]; *p* < 0.001), and depressive symptoms (11.0 [IQR: 9]; 6.0 [IQR: 5.75]; *p* = 0.002). For pain catastrophizing, significant differences were observed in the rumination (7.0 [IQR: 8.8]; 4.5 [IQR: 5.75]; *p* = 0.047) and helplessness (7.0 [IQR: 7.0]; 5.0 [IQR: 6.0]; *p* = 0.050) subscales, while magnification scores did not differ significantly (*p* = 0.134), the total PCS score were also elevated in the anxiety group (20.0 [IQR: 13.5]; 13.0 [IQR: 12.75]; *p* = 0.032).

### 3.4 Lifestyle Variables

No significant differences were found between groups in terms of physical activity (*p* = 0.770), social activity (*p* = 0.606), coffee consumption (*p* = 0.537), alcohol use (*p* = 0.417), or smoking status (*p* = 0.225).

### 3.5 Correlations of Assessed Variables in Anxiety Groups

**FIGURE 1:**
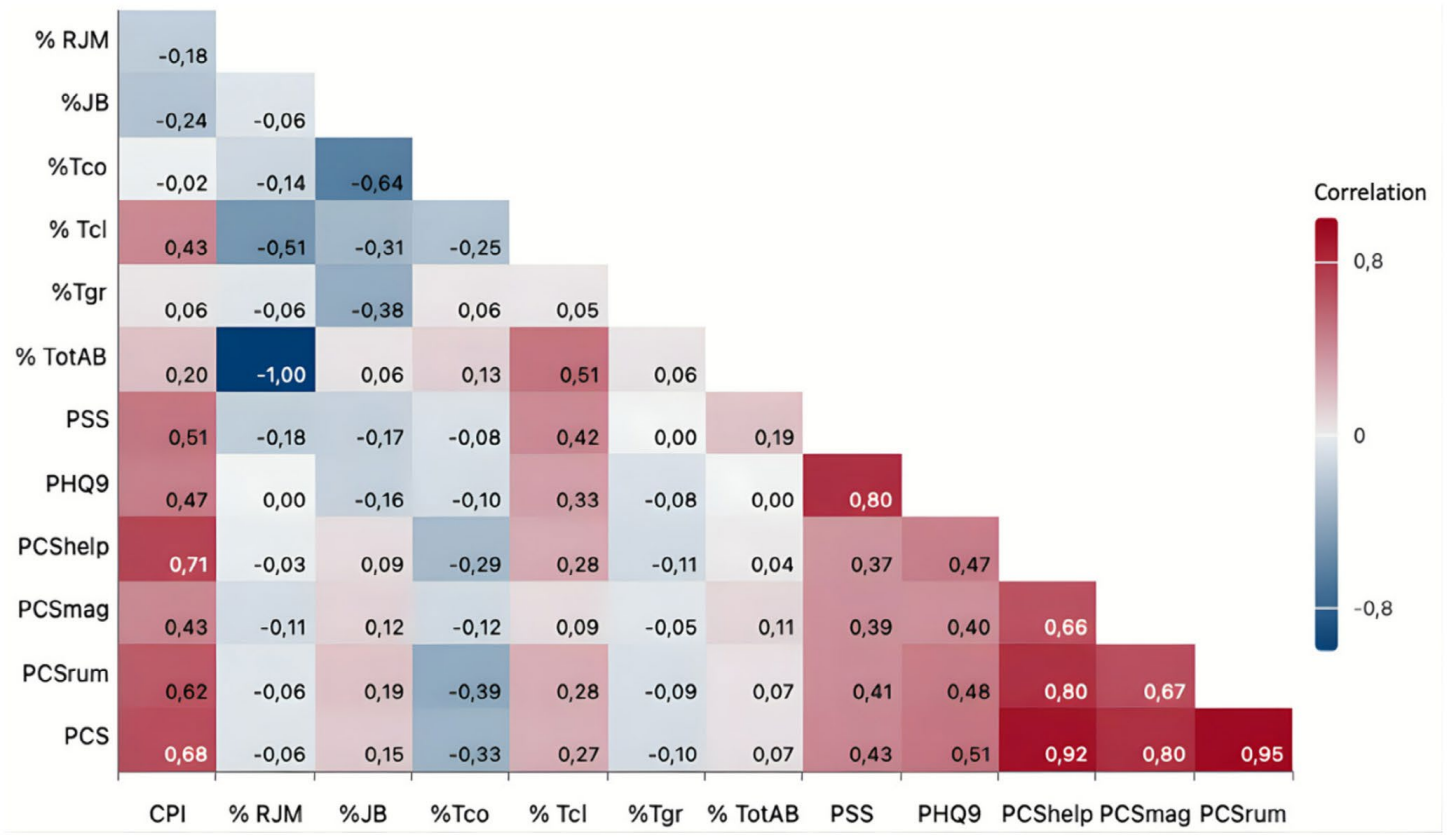
Spearman correlation heat map, psychological distress and bruxism behaviours in the TMD Patients with elevated symptoms of anxiety. Correlation coefficients (*ρ*) are represented by the colour intensity. Positive correlations appear in blue, negative in red. Only statistically significant correlations (*p* < 0.05) are shown. JB, Jaw Bracing; PCS, Pain Catastrophizing Scale; PCShelp, helplessness; PCSmag, magnification; PCSrum, rumination; PHQ‐9, Patient Health Questionnaire–9; PSS, Perceived Scale Stress; RJM, Relaxed Jaw Muscle; Tcl, Teeth Clenching; Tco, Teeth Contact; Tgr, Teeth Grinding.

Correlation analyses in the group with elevated symptoms of anxiety revealed moderate to strong positive associations between CPI and psychological variables (Figure 1), including perceived stress (PSS, *ρ* = 0.518, *p* < 0.001), depression (PHQ‐9, *ρ* = 0.470, *p* = 0.001), and pain catastrophizing (PCShelplessness, *ρ* = 0.715; PCSmagnification, *ρ* = 0.430; PCSrumination, *ρ* = 0.620; total PCS, *ρ* = 0.688; all *p* < 0.001). Additionally, PSS (*ρ* = 0.424, *p* = 0.005) and CPI (*ρ* = 0.430, *p* = 0.029) were moderately correlated with tooth clenching frequency (Table 2). The normative anxiety group did not exhibit any significant correlations.

**TABLE 2:** Spearman correlation between AB behaviours and psychological examined variables in the TMD Patient with elevated symptoms of anxiety.

Variable 1	Variable 2	Rho	*p*
PSS	CPI	0.518^a^	< 0.001
PHQ9	CPI	0.470^a^	< 0.001
PCShelp	CPI	0.715^b^	< 0.001
PCSmag	CPI	0.430^a^	< 0.001
PCS rum	CPI	0.620^b^	< 0.001
PCS	CPI	0.688^b^	< 0.001
Alcohol	CPI	0.407^a^	0.039
PSS	% Tooth Clenching	0.424^a^	0.005
CPI	%Tooth Clenching	0.430^a^	0.029

*Note:* Sig. (*p* < 0.05).

^a^ Moderate correlation.

^b^ Strong correlation.

## 4 Discussion

This study provides novel insights into the role of anxiety in the interplay between psychological variables and real‐time AB behaviours through the EMA assessment in patients with painful TMD. The main findings revealed that individuals with clinically elevated symptoms of anxiety exhibited significantly higher frequencies of AB behaviours, characterised principally by tooth clenching and increased psychological distress, including elevated stress perception, catastrophizing and depressive symptoms. Notably, these patients also reported greater clinical pain intensity, which showed positive moderate and strong correlations with all psychological variables. These results underscore the relevance of anxiety‐related mechanisms in shaping both the perception and behavioural expression of AB bruxism and pain in TMD patients, contributing to a deeper understanding of individual vulnerability profiles.

Despite similar demographic features such as age and lifestyle, the elevated symptoms anxiety group presented higher levels of pain intensity (CPI). Additionally, several moderate and stronger correlations were presented between CPI and psychosocial variables. The interplay between these variables suggests that emotional dysregulation, especially anxiety, may exacerbate central sensitization leading to increased pain perception. This aligns with data from the OPPERA cohort, which highlighted that psychosocial variables, rather than sociodemographic features, are the strongest predictors of TMD onset and persistence [11, 35]. Recent population‐based data further supports this view, demonstrating that anxiety and depression are more strongly associated with TMD severity, primarily reflected by pain intensity than age or lifestyle habits. Thus, our findings reinforce the central role of anxiety in modulating both pain and masticatory activity in TMD. Notably, in the present study, this elevated anxiety group also comprised a higher proportion of women (83.3% vs. 54.8%; *p* = 0.011), aligning with evidence that females are more susceptible to both anxiety disorders and painful TMD [36, 37]. An odds ratio of 0.242 (95% CI: 0.077–0.75) indicates that females were over four times more likely to exhibit clinically significant anxiety symptoms than men. This sex‐based vulnerability may be partially explained by hormonal influences, as oestrogen and related hormones can increase pain sensitivity by enhancing temporal summation and reducing endogenous inhibition in women [38, 39]. Moreover, the interaction between increased emotional sensitivity and a reduced capacity for descending pain modulation may have led these anxious female patients particularly susceptible to experience greater pain intensity [40]. These findings highlight the role of emotional dysregulation, especially in women, in amplifying pain processing and contributing to a more severe clinical phenotype in TMD.

AB behaviours through EMA revealed marked differences in muscle activity patterns. Patients with clinically elevated symptoms of anxiety group showed a higher frequency of tooth clenching compared with the normative anxiety group, suggesting that anxiety may potentiate sustained masticatory muscle activity. Individuals with clinically elevated symptoms of anxiety group may show greater muscle spindle excitability and reduced cortical inhibition, predisposing them to sustained jaw contraction under stress. This reflects a maladaptive feedback loop where emotional hyperarousal increases muscle tension [12]. Additionally, tooth clenching showed a moderate correlation with CPI in the elevated symptoms of anxiety group, suggesting that this specific AB behaviour may contribute to the development or maintenance of clinical pain in these individuals. The higher total AB frequency observed in this group further supports the notion of a sensitised motor system primed for activation under psychological distress.

Regarding the psychosocial variables, perceived stress was markedly higher in the clinically elevated symptoms of anxiety group, supporting the well‐established bidirectional relationship between anxiety and stress in pain conditions. Additionally, in this study, perceived stress showed a moderate positive correlation with tooth clenching behaviour (*R* = 0.47, *p* < 0.001). These findings reinforce the hypothesis that psychological stress contributes to the initiation and maintenance of AB through dysregulation of autonomic and central mechanisms; however, under the influence of anxiety symptoms. Stress may heighten vigilance towards somatic sensations and reduce descending pain inhibition, thereby amplifying muscle tension and parafunctional motor output [41]. Furthermore, stress‐induced activation of the hypothalamic–pituitary–adrenal axis and sympathetic nervous system has been linked to enhanced excitability of trigeminal nociceptive pathways, contributing to increased clenching behaviour [42]. Taken together, these mechanisms suggest that the detrimental impact of stress on AB is amplified when co‐occurring with elevated anxiety symptoms [43]. This interaction is supported by evidence indicating that anxiety and stress share overlapping neurobiological pathways [44], with anxiety heightening stress reactivity and prolonged stress further exacerbating anxiety, thereby establishing a bidirectional cycle that sustains muscle activity and painful TMD [45].

Pain catastrophizing also emerged as a relevant psychological mechanism closely associated with anxiety. Individuals with clinically elevated symptoms of anxiety group scored significantly higher values than the normative anxiety group on the helplessness and rumination subscales, as well as on the total PCS score, suggesting that heightened anxiety may predispose individuals to more frequent catastrophic thinking. Evidence indicates that catastrophizing is a strong predictor of pain severity, emotional distress and treatment outcomes in chronic pain populations [46]. Rumination may serve as a cognitive anchor that sustains anticipatory worry and somatic hyperawareness [47]. In anxious individuals, this persistent focus on bodily sensations and perceived threats may heighten masticatory muscle reactivity, promoting sustained behaviours like clenching, even in the absence of external stressors, as an implicit yet ineffective strategy to regain internal control [47]. Likewise, depressive symptoms were also significantly higher in the clinically elevated symptoms of anxiety group. Depression has been associated with altered central pain modulation and impaired emotional resilience [48]; the co‐occurrence of anxiety and depression may contribute and potentiate dysregulation of pain processing networks. Viewed together, this compounded psychological load not only amplifies pain perception but also reduces treatment responsiveness, particularly in TMD subtypes characterised by muscular hyperactivity [4]. Crucially, while each psychological construct, catastrophizing, rumination, helplessness, depression, has independent relevance, it is their interactive and cumulative effect, often rooted in heightened anxiety, that sustains both central sensitization and AB behaviours [3, 49, 50].

The convergence of elevated anxiety, increased AB activity, higher perceived stress, greater catastrophizing and depression in our TMD cohort supports a mechanistic model in which emotional dysregulation acts as both a trigger and amplifier of TMD. Importantly, patients presenting this profile of affective vulnerabilities also reported the highest frequencies of AB behaviours, especially clenching, indicating that these observable motor patterns may act as expressions of psychological distress. These findings reinforce the importance of assessing and including psychological factors in the therapies for AB behaviours and in targeting AB behaviours not only as potential contributors to symptoms, but also as modifiable risk factors that may actively contribute to the development and maintenance of painful TMD. Multidisciplinary treatment approaches, encompassing behavioural regulation, emotional coping and muscle relaxation strategies, should therefore be prioritised in this high‐risk subgroup. Future longitudinal studies are needed to determine causal relationships and assess the efficacy of targeted psychological therapies, such as cognitive‐behavioural approaches, in reducing AB frequency and improving pain outcomes.

Lifestyle factors, including physical activity, social engagement, alcohol and tobacco use, did not differ significantly between groups. Although these variables have been associated with pain and AB in previous literature, their limited influence in this study may reflect the predominance of psychological drivers in this specific clinical population [51, 52]. Nonetheless, lifestyle variables should continue to be monitored in longitudinal contexts, as their cumulative or indirect effects may become more evident over time.

It is important to mention that the present study is strengthened by the application of EMA methodology, which captures AB behaviours in real time and thereby reduces recall bias while enhancing ecological validity. The integration of comprehensive psychological assessments with behavioural outcomes provides a multidimensional perspective that strengthens the interpretation of our findings. However, the cross‐sectional design prevents causal inferences, and the sample size, though clinically relevant, may restrict broader generalizability. Moreover, reliance on self‐report instruments and the absence of objective physiological markers (e.g., EMG, cortisol) represent limitations that should be addressed in future longitudinal investigations.

## 5 Conclusion

This study demonstrated that anxiety plays a key role in the expression of AB behaviours and the psychosocial profile of patients with painful TMD. Notably, tooth clenching emerged as the most characteristic behaviour in painful TMD patients with elevated anxiety symptoms. Therefore, elevated anxiety symptoms should be considered a clinical target in the management of AB behaviours and pain in TMD patients.

## Author Contributions

This study was designed by D.M.F., J.S.‐B. and P.C.R.C. The data collection was performed by D.M.F. The data were analysed and interpreted by D.M.F. The results were critically examined by all authors. D.M.F. had a primary role in preparing the manuscript, which was edited by D.M.F, G.D.C. and S.P.B. All authors have approved the final version of the manuscript and agree to be accountable for all aspects of the work.

## Funding

This study was supported by the Programa de Excelência Acadêmica (PROEX) from the Coordenação de Aperfeiçoamento de Pessoal de Nível Superior (CAPES), Brazil.

## Conflicts of Interest

The authors declare no conflicts of interest.

## Data Availability

The datasets generated during and/or analysed during the current study are not publicly available but are available from the corresponding author upon reasonable request and completion of an institutional data transfer agreement.
